# Repercussions of the second victim phenomenon on health care workers: Scoping review

**DOI:** 10.1371/journal.pone.0357203

**Published:** 2026-09-15

**Authors:** Elaine Cristine da Conceição Vianna Gonçalves da Costa, Luana Cardoso Pestana, Laríssia Admá de Souza Pereira, Fabrício Glauber Suzano Maciel, Renata Eloah de Lucena Ferretti-Rebustini, Olga Maria Pimenta Lopes Ribeiro, Cíntia Silva Fassarella, Cristiane Helena Gallasch

**Affiliations:** 1 Postgraduate Program in Nursing, State Universidade do Rio de Janeiro, Rio de Janeiro, Brazil; 2 Medicinal Nursing Department, Graduate Program on Adult Health Nursing, University of São Paulo School of Nursing, São Paulo, Brazil; 3 Nursing School of the University of Porto, Porto, Portugal; Universiti Sains Malaysia - Kampus Kesihatan, MALAYSIA

## Abstract

Patient safety is a key pillar of healthcare services and the quality of care provided. Despite ongoing efforts, safety incidents still occur, resulting in victims beyond the patient. The healthcare professionals involved are considered second victims, warranting studies in the field of worker safety.

**Objective:**

To map the repercussions experienced by healthcare professionals who have experienced the “second victim” phenomenon resulting from patient safety incidents in healthcare services.

**Method:**

A scoping review guided by the JBI and PRISMA-ScR methodologies. A preliminary search was conducted, finding no evidence of existing records or publications with the same objective. The protocol was registered on the Open Science Foundation platform. The search strategy followed the three recommended steps. The databases consulted were MEDLINE, SCOPUS, CINAHL, WOS, LILACS, and, for gray literature, the Brazilian Digital Library of Theses and Dissertations and ProQuest. There were no restrictions on language, year, or methodology; the PCC mnemonic was used as an eligibility criterion: population—healthcare professionals; concept—second victim phenomenon related to patient safety incidents; context—healthcare services. Studies that did not meet the objective and PCC criteria were excluded. The selection and data extraction were performed by independent researchers and conducted anonymously.

**Results:**

Of the 2,129 records retrieved, 37 publications comprised the final sample. The publications were collected between 2009 and 2024 and were identified in 22 countries across five continents. There was a predominance of publications from Europe (n = 12), with qualitative studies being the most common (n = 14); the hospital setting was the most frequently cited (n = 25), and nursing was the most studied population (n = 27). The most frequently cited health descriptors were “second victims” (n = 17) and “adverse events” (n = 14). Of the selected studies, 13 used measurement instruments to identify the phenomenon. Thirty-nine repercussions were mapped, categorized from the perspective of worker health in physical, mental, professional and social dimensions based on the study’s assumptions. The professional dimension presented the highest number of repercussions. The mental dimension identified suicidal thoughts or behavio as a risk to physical integrity.

**Conclusion:**

The repercussions indicate a strong relationship between the second victim phenomenon and worker health and safety, impacting the professional and personal lives of the professionals involved.

## Introduction

Patient safety is a strategic priority and a matter of global concern in the field of healthcare; however, despite advances in policies and actions, safety incidents still occur, resulting in victims beyond the patients themselves [1,2].

In Brazil, 426.079 incidents and adverse events were reported in 2024 [3], highlighting the magnitude of the challenge related to patient safety and reinforcing the need to implement effective strategies for the reduction and elimination of preventable harm, in line with the guidelines established by the Global Action Plan for Patient Safety 2021–2030 [2].

Safety incidents are defined as events or circumstances that could have resulted (near miss) or did result in unnecessary harm to the patient, and are classified into three categories: *near miss*, when the patient is not harmed; incident without harm, where the event affected the patient but did not cause discernible harm; and incident with harm, where the event resulted in harm to the patient, referred to as an adverse event [4].

Undoubtedly, patient safety incidents, regardless of whether or not they result in harm, can be devastating and directly affect those receiving care, known as the primary victims. However, their impacts are not limited to patients; they also affect the healthcare professionals involved in these incidents, who experience significant emotional, psychological, and professional repercussions, and are recognized in the literature as “second victims” [2,5].

The term “second victim” was first used in 2000 to describe medical professionals who made mistakes and became victims of the event [6]. The concept was expanded in 2009 to include all healthcare professionals who experience psychological and professional distress following an adverse event, a medical error, or an unforeseen injury caused to a patient [7].

Although the term faced harsh considerations [8] only after the publication of the initial concept [6] and the proposal to expand it [7], the distress experienced by healthcare professionals resulting from their involvement in healthcare safety incidents has been well documented [6–9].

In response to questions raised by researchers opposed to the use of the term [8], the European network of researchers working with second victims (ERNST) revised the concept [10]. From this perspective, it was understood that without reducing the liability of the professionals involved or diminishing recognition of the harm suffered by the primary victims, healthcare professionals also suffer from the consequences of safety incidents [11,12].

Thus, the review of the concept defined a “second victim” as any healthcare worker who is directly or indirectly involved in an unexpected event for the patient, consisting of an unintentional care error or an injury resulting from care, thereby also becoming a victim to the extent that they suffer the negative impacts [10].

The phenomenon of the second victim exhibits characteristics that can be classified as acute stress disorder or post-traumatic stress disorder, encompassing physical repercussions such as tachycardia, insomnia, fatigue, loss of appetite, and muscle pain, as well as psychological repercussions such as feelings of sadness, guilt, fear, shame, anxiety, anger, and frustration [2,5–8,10,12,13].

It should be noted that acute stress disorder is characterized by transient symptoms that emerge in the first few days following the event and last for a short period, no longer than one month, and resolve with or without external support. In contrast, more enduring reactions with a prolonged response are recognized as post-traumatic stress disorder [13,14].

Post-traumatic stress disorder leads to behavioral changes that significantly affect the daily lives of professionals, who begin to experience social withdrawal, depersonalization, rumination on the event, and avoidance of places or situations that cause them to relive the incident [2,5–8,10,13].

In addition to these repercussions, the phenomenon carries legal consequences in both civil and criminal spheres, potentially leading to the loss of professional licenses and employment, a fact that intensifies suffering [13,14]. The phenomenon of the second victim, being intense and multifaceted, has an intrinsic relationship with occupational safety regarding the provision of healthcare [5,8,11].

Thus, given the interconnection between patient safety and the safety of healthcare professionals, this review aimed to map the repercussions experienced by healthcare professionals who have experienced the “second victim” phenomenon resulting from patient safety incidents in healthcare settings [1,2,8,10].

## Method

This is a scoping review, guided by the JBI methodology and the *Preferred Reporting Items for Systematic Reviews and Meta-Analyses* (PRISMA-ScR) tool for scientific writing [15,16].

Initially, a search was conducted on the Open Science Framework and JBI platforms; no manuscripts or protocol records with an objective similar to that of this review were found.

The protocol for this scoping review followed the *Preferred Reporting Items for Systematic Review and Meta-Analysis Protocols* (PRISMA-P) [17], was registered on the *Open Science* Framework [18], and subsequently published in an internationally circulated scientific journal [19].

The Preferred Reporting Items for Systematic Reviews and Meta-Analyses: Scope Reviews (PRISMA-ScR) checklist is included in the supplementary material.

To guide the formulation of the review question, the PCC mnemonic [20] was used, where the population (P) was healthcare professionals, the concept (C) was the second victim phenomenon resulting from patient safety incidents, and the context (C) was healthcare services. The established review question was: ***What does the literature reveal about the repercussions experienced by healthcare professionals who have experienced the second victim phenomenon resulting from patient safety incidents in healthcare services?***

### Eligibility and exclusion criteria

In accordance with JBI recommendations, no restrictions were applied regarding study type, methodological approach, publication period, or language [20].

We included primary research studies, review articles, and editorials that addressed the impact of the “second victim” phenomenon on healthcare professionals. The publications were read and evaluated by reviewers proficient in English and Spanish. For publications in other languages, the reviewers used Google Translate to assist in interpreting the content.

Exclusion criteria included publications that addressed exclusively students in regulated or unregulated health professions and those that did not meet the inclusion criteria or the objective of this review.

The review team evaluated the titles, abstracts, and full texts, and the report explaining why each record was excluded is available on the OSF platform.

### Search strategy

The search strategy followed the three steps proposed by the JBI, with the first step conducted on July 22, 2024, through a preliminary search in the Medical Literature Analysis and Retrieval System Online (MEDLINE) database, using the search terms “Second Victim Phenomenon,” “Health Personnel,” and “Health Services.” As a result, 292 publications were retrieved. On January 8, 2025, this stage was updated, retrieving 371 publications.

In the second stage, which took place on January 10, 2025, a literature review was conducted in the MEDLINE, SCOPUS, Web of Science (WOS), CINAHL, and Latin American and Caribbean Health Sciences Literature (LILACS) databases, using the search strategy presented in Table 1. It should be noted that the refinement of the strategy for each information source consulted involved a librarian with experience in systematic reviews. In addition to the databases, a search of the gray literature was also conducted through the Brazilian Digital Library of Theses and Dissertations (BDTD) and the PROQUEST platform.

**Table 1:** Search strategy for the retrieval of records from databases and gray literature. Rio de Janeiro, RJ, Brazil, 2025.

Search strategies		n
**MEDLINE**	(Second Victim*[tiab]) AND (Health Personnel[mh] OR Health Services[mh] OR Hospitals[mh] OR Health[tiab] OR Healthcare[tiab] OR Hospital*[tiab]) AND (“2024/06/27”[PDAT]: “2025/01/07”[PDAT])	468
**SCOPUS**	TITLE-ABS-KEY (“Second Victim*”) AND TITLE-ABS(“Health Personnel” OR “Health Services” OR Health OR Healthcare OR Hospital*) AND (LIMIT-TO(PUBYEAR, 2024) OR LIMIT-TO(PUBYEAR, 2025))	457
**WOS**	TS=(“Second Victim*”) AND TS=(“Health Personnel” OR “Health Services” OR Health OR Healthcare OR Hospital*) AND PY=(2024 OR 2025)	381
**CINAHL**	(“Second Victim” OR “Second Victims” OR “Segunda Vitima”) AND (“Health Personnel” OR “Health Services” OR Health OR Healthcare OR Hospital* OR Saude)	208
**LILACS**	(“Second Victim” OR “Second Victims” OR “Segunda Vitima” OR “Segunda Victima”) AND (“Health Personnel” OR “Health Services” OR Health OR Healthcare OR Hospital* OR Saude OR Salud) AND (db:(“LILACS”)) AND (year_cluster:[2024–2025])	22
**BDTD**	(“Second Victim” OR “Second Victims” OR “Segunda Vitima”)	08
**PROQUEST**	(“Second Victim” OR “Second Victims”) AND (“Health Personnel” OR “Health Services” OR Health OR Healthcare)	585

Source: The authors, Rio de Janeiro, Brazil, 2025.

In the third stage of the search strategy, we manually reviewed the reference lists of the publications selected after full-text screening, following the selection and screening protocol recommended for systematic reviews.

### Study selection

Study selection was conducted by the principal investigator, who performed a pilot test to evaluate 25 titles and initial abstracts with the selection team. In this round, agreement among reviewers exceeded 75%, allowing the review to proceed based on the eligibility criteria.

The studies identified as relevant were screened by two independent reviewers in an anonymized manner (ECCV and LCP). The 65 discrepancies found during the selection process were resolved by consensus among the reviewers, with the involvement of a third reviewer (LA) for the final decision.

The reviewers mapped the data independently, discussing the results at each stage and continuously updating the data extraction form interactively, as recommended by the method.

### Data extraction

For data extraction, the reviewers followed the customized form template, which included the authors’ names, publication title, year, country where the research was conducted, publication type, objective, study design, population, study setting, identified repercussions, tools for identifying the phenomenon, and keywords or descriptors used in the publications.

### Analysis of the repercussions resulting from the second victim phenomenon

The data were extracted through a script previously prepared in the review protocol, in which the physical, mental, professional, social and psychosocial dimensions were defined for the initial grouping of the repercussions.

During the thematic analysis, the category initially called psychosocial was discontinued due to the conceptual overlap with the mental, professional and social dimensions, which compromised the analytical delimitation between the categories. Thus, their findings were redistributed according to the predominant manifestation of each repercussion.

The reorganization of the results into four dimensions compatible with the way the theoretical frameworks used understand the work-related health-disease process. Thus, the physical, mental, professional and social dimensions allowed analytical consistency and better alignment with the adopted theoretical frameworks, which understand work-related illness as a multidimensional process.

The interpretation of the findings was based on the Psychodynamics of Work, which understands health problems as produced in the interaction between the organization of work, socio-professional relations and the processes of subjectivation, and on the Social Determinants of Health, according to which working conditions are an important determinant of workers' health and illness.

The analysis was carried out independently by the members of the review team and the divergences were resolved in a consensual manner based on the theories used to support the analysis.

## Results

**Fig 1:**
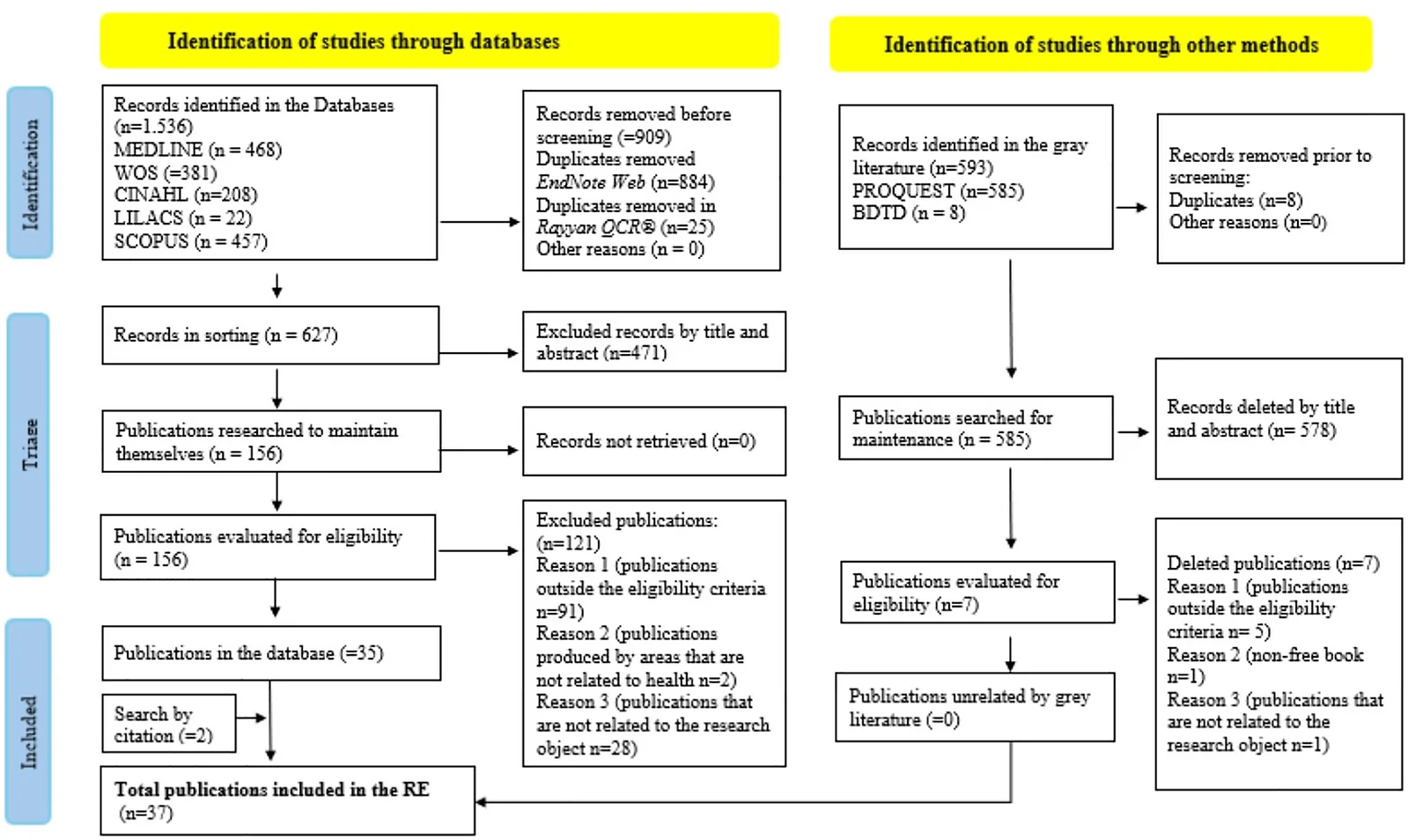
PRISMA SCR of identification, selection, and inclusion of studies containing variables related to the repercussion experienced by health professionals resulting from the phenomenon of second victim, Rio de Janeiro, RJ, Brazil, 2025. Source: Adapted from AROMATARIS et. al., 2014 and TRICCO, 2018.

After conducting a search of the databases and gray literature, 2,129 records were retrieved following the screening and selection stages, as shown in the PRISMA SCR flowTable illustrated in Fig 1.

EndNote Web^®^and Rayyan QCR® software were used to remove duplicates and evaluate titles and abstracts.

After applying the eligibility criteria and following all steps recommended by the JBI, 37 publications were included in the final sample of the scoping review. A summary of the selected studies is presented in Table 2.

**Table 2:** Synthesis of the studies included in the scoping review with characterization of identification, year, origin, objective, study design, population, and health service. Rio de Janeiro, RJ, Brazil, 2025 (more).

Author	Manuscript title	Country	Objective	Study design	Population	Scenario
Scott SD et al, 2009 [6]	*The natural history of recovery for the healthcare provider "second victim" following adverse patient events*	England	To describe and characterize the experiences and recovery trajectories of previous victims.	Qualitative	Healthcare professionals	Not cited
Seys D et al., 2013 [21]	*Health care professionals as second victims after adverse events: a systematic review*	Belgium	To determine the definitions of this concept, investigate the prevalence and impact of the adverse event on the second victim, and the coping strategies used.	Systematic review	Health care professionals Second victims of medical errors	Not cited
Ullström et al., 2014 [22]	*Suffering in silence: a qualitative study of second victims of adverse events*	Sweden	To investigate how healthcare professionals at a Swedish university hospital were affected by their involvement in adverse events, with an emphasis on the organizational support they needed and the organizational support they received.	Cross-sectional	Healthcare professionals	Hospitals
Grissinger M, 2014 [23]	*Too Many Abandon the “Second Victims” of Medical Errors*	United States	Describing the suffering and impact of second victims	Editorial	Healthcare professionals	Various scenarios
Harrison Reema et al., 2015 [24]	*Emotion and Coping in the Wake of Medical Error: A Cross-Country Study*	England	To investigate the following: a) the professional or personal disruption experienced after committing an error, b) the emotional response and coping strategies used, c) the relationship between emotions and the selection of coping strategies, d) factors influencing clinicians’ responses, and e) perceptions of organizational support.	Cross-sectional	Physicians and nurses	Hospital
Mira JJ et al., 2015 [25]	*The aftermath of adverse events in Spanish primary care and hospital health professionals*	Spain	Assessing the impact of adverse events in primary care and hospitals in Spain on second victims	Cross-sectional	Doctors and nurses	Hospitals and health centers
Chan ST et al., 2017 [26]	*Psychological responses, coping, and support needs of healthcare professionals as second victims*	Singapore	To provide an overview of psychological responses, coping strategies, and support needs	Systematic review	Healthcare professionals involved in adverse events	Various healthcare departments
Serou N, 2017 [27]	*Systematic review of psychological, emotional, and behavioral impacts of surgical incidents on operating room staff*	England	To explore the effect of surgical incidents on operating room staff and their subsequent behaviors.	Systematic review	Healthcare professionals Operating room staff	Hospitals, operating rooms
Kable A et al., 2018 [28]	*Effects of adverse events in healthcare on acute care nurses in an Australian context: A qualitative study*	Australia	Understanding the effects of adverse events in healthcare on nurses in acute care settings in an Australian context	Qualitative	Nurses	Hospitals
Amjad Mohamadi-Bolbanabad et al., 2019 [29]	*The second victims’ experience and related factors among medical staff*	Iran	To determine the experience of second victims and related factors among medical staff	Cross-sectional	Doctors, nurses, and midwives	Public hospitals
Vanhaecht K et al., 2019 [30]	*Duration of second victim symptoms in the aftermath of a patient safety incident and association with the level of patient harm: a cross-sectional study in the Netherlands*	Belgium	To describe the prevalence and duration of self-reported symptoms among healthcare professionals triggered by the PSI and the association between these symptoms and the degree of patient harm caused by the incident.	Cross-sectional	Healthcare professionals	Hospitals
Biggs S et al., 2020 [31]	*Impact of surgical complications on the operating surgeon*	England	Surgeons’ experience with serious surgical complications	Cross-sectional	Physicians Surgeons of all specialties	Hospital
Busch IM et al., 2020 [32]	*Psychological and Psychosomatic Symptoms of Second Victims of Adverse Events: a Systematic Review and Meta-Analysis*	Italy	Systematically reviewed the types and prevalence of psychological and psychosomatic symptoms among second victims.	Systematic review	Healthcare professionals involved in adverse events	Not mentioned
Choi EY et al., 2020 [33]	*Nurses' experiences of patient safety incidents in Korea: a cross-sectional study*	South Korea	To determine the experiences and impacts of the second victim phenomenon among Korean nurses in various aspects	Qualitative	Nurses	Hospitals
Pyo J et al., 2020 [34]	*Physicians' Difficulties Due to Patient Safety Incidents in Korea: a Cross-Sectional Study*	South Korea	To quantitatively examine the characteristics of physicians’ experiences with PSIs, along with the resulting difficulties and levels of post-traumatic stress disorder (PTSD) and post-traumatic grief disorder (PTGD) in relation to the events	Quantitative	Physicians	Not cited
White RM et al., 2020 [35]	*Second victim phenomenon: Is 'just culture' a reality? An integrative review*	United States	To explore the second victim phenomenon and the adoption of "just culture" in healthcare institutions since the publication of the historical systematic review by Seys et al. in 2010	Integrative review	Healthcare professionals	Various care settings
Buhlmann M et al., 2021 [36]	*The impact of critical incidents on nurses and midwives: A systematic review*	Australia	To examine international research and develop a contemporary understanding of the impact that critical incidents have on nurses and midwives	Systematic review	Nurses and midwives	Not cited
Burlison J et al., 2021 [37]	*The Effects of the Second Victim Phenomenon on Work-Related Outcomes: Connecting Self-Reported Caregiver Distress to Turnover Intentions and Absenteeism*	United States	To assess the relationships between self-reported second victim distress and turnover intentions and absenteeism	Quantitative	Nursing staff	Pediatric hospital
Luk LA et al., 2021 [38]	*Healthcare Professional Experiences of Clinical Incidents in Hong Kong: A Qualitative Study*	Hong Kong	To explore healthcare professionals’ experiences with clinical incidents, their impacts, and needs	Mixed-methods research	Healthcare professionals Physicians, nurses, and allied health staff	Not cited
Schiess Cornel et al., 2021 [39]	*The Transactional "Second-Victim" Model—Experiences of Affected Healthcare Professionals in Acute-Somatic Inpatient Settings: A Qualitative Metasynthesis*	Australia	To identify, describe, and interpret these experiences in inpatient settings with patients with acute somatic syndrome.	Qualitative metasynthesis	Healthcare professionals	Hospitals—inpatient and intensive care settings
Stovall, M., et al., 2021 [40]	*Suicide Risk, Changing Jobs, or Leaving the Nursing Profession in the Aftermath of a Patient Safety Incident*	United States	To examine the relationship between nurses’ sociodemographic data and our study’s unique variables with potentially morally harmful outcomes (i.e., the study variables of leaving the profession: changing jobs, intention to leave the profession, or suicidal ideation).	Qualitative	Nurses	Hospitals
Tanabe K et al., 2021 [41]	*Caring for the caregiver following an adverse event*	United States	Review the potential for adverse impacts on healthcare professionals following a medical error or unfavorable clinical outcome	Qualitative Case study	Physician	Hospital - operating room
Bakshi A. S et al., 2022 [42]	*Second Victim Syndrome in Trauma Practitioners and Other Ancillary Staff*	United States	To assess second victim syndrome and its sequelae in trauma care providers and support staff	Qualitative	Healthcare professionals Physicians, nurses, advanced practice providers, dietitians, occupational therapists, social workers	Hospital - Urban-level trauma center
Ben Saida et al., 2022 [43]	*North African doctors as second victims of medical errors: a cross-sectional survey on the treatment of patients*	Tunisia	To describe the profile, types, and severity of medical errors and explore the psychological impact on “second victims” to better understand how they cope with the situation.	Cross-sectional	Senior and junior physicians	Hospital
Ganahl S et al., 2022 [44]	*Second Victims in Intensive Care—Emotional Stress and Traumatization of Intensive Care Nurses in Western Austria after Adverse Events during the Treatment of Patients*	Austria	To assess the natural history and cause of second victim traumatization	Qualitative	Nurses	Hospital - intensive care
Jain G. et al., 2022 [45]	*"Second Victim" Syndrome Among Surgeons from South Asia*	India	To assess its prevalence and impact among surgeons from South Asia	Qualitative	Surgeons	Hospitals
Balogun et al., 2023 [46]	*Surgical residents as "second victims" following exposure to medical errors in a tertiary health training facility in Nigeria: a phenomenological study*	Nigeria	Identify the prevalence of the second victim phenomenon among residents, determine the impact of the event, identify strategies, and explore improvements	Qualitative Phenomenological	Surgical residents	Hospital
Cohen R et al., 2023 [47]	*Nurses' Silence: Understanding the Impacts of the Second Victim Phenomenon among Israeli Nurses*	Israel	To use the natural history model of second victim recovery to examine the impact of the phenomenon on Israeli nurses, with a specific focus on the organizational support they felt they needed compared to the support they felt they received from their organizations.	Qualitative	Nurses who experienced second victim syndrome	Various healthcare departments
Kappes M et al., 2023 [48]	*Prevalence of the second victim phenomenon among intensive care unit nurses and the support provided by their organizations*	Chile	To determine the prevalence of the second victim phenomenon, focusing on psychological distress, among Chilean adult ICU nurses and its relationship to their perception of organizational support.	Quantitative	Nurses	Hospitals—intensive care
Mousa O et al., 2023 [49]	*A Study on Patient Safety Incidents and the Second Victim Phenomenon Among Healthcare Providers in Al-Ahsa, Saudi Arabia*	Saudi Arabia	To examine the second victim phenomenon among healthcare providers in Al-Ahsa hospitals, its prevalence, symptoms, associated factors, and support strategies.	Cross-sectional	Healthcare providers	Hospitals
O'Meara S et al., 2023 [50]	The psychological impact of adverse events on urology trainees	Ireland	To collect data on the emotional impact and potential second victim syndrome in relation to adverse events with surgeons practicing in the Republic of Ireland.	Qualitative	Resident surgeons in urology	Hospitals
Sachs CJ et al., 2023 [51]	*Second Victim Syndrome*	United States	To describe the risk factors, symptoms, assessment, and management of second victim syndrome	Qualitative descriptive	Healthcare professionals	Not cited
Silveira Sibele et al., 2023 [52]	*Impacts of patient safety incidents in nursing: a look at the second victim*	Brazil	To understand the impacts on nursing professionals as the second victim of patient safety incidents.	Qualitative	Nurses	University hospital
Brunelli MV et al., 2024 [53]	*Second Victim Experience: A Dynamic Process Conditioned by the Environment. A Qualitative Research*	Argentina	Exploring the experiences of second victims among healthcare professionals in Argentina	Qualitative Phenomenological	Healthcare professionals involved in adverse events	Hospital
Chong RIH et al., 2024 [54]	*Scoping review of the second victim syndrome among surgeons: Understanding the impact, responses, and support systems*	Singapore	To consolidate existing studies regarding a surgeon’s experience with SVS, broadly examining prevalence and impact, identifying types of responses, and assessing factors that could influence them.	Scoping review	Surgeons	Hospitals
Mahat S et al., 2024 [55]	*Impact of second victim distress on healthcare professionals' intent to leave, absenteeism, and resilience: A mediation model of organizational support*	Finland	To examine the relationship between second victim distress and the outcome variables, specifically: "intentions to leave, absenteeism, and resilience." Additionally, this study also assessed how organizational support mediates the relationship between second victim distress and the outcome variables.	Quantitative	Nurses and physicians	Hospitals
Yaow CYL et al., 2024 [56]	*Intraoperative adverse events among surgeons in Singapore: a multicenter cross-sectional study on impact and support*	Singapore	To explore the extent to which the experience of an adverse event is common among surgeons in Singapore, as well as its impact and the factors affecting their responses and perceived support systems.	Cross-sectional	Surgeon	Hospitals

Elaborated by authors, 2025.

In mapping the repercussions related to the second victim phenomenon, the publications were further analyzed regarding methodological design, geographic distribution, identified professional categories, practice settings, temporal distribution, instruments for identifying the phenomenon, and adherence to health descriptors used in the publications included in the review, as described below:

### Methodological profile of the studies

The studies were categorized according to the nomenclature adopted by the authors; as a result, the search identified 14 qualitative studies, nine cross-sectional studies, five systematic reviews, four quantitative studies, one mixed-methods study, one qualitative meta-synthesis, one integrative review, and one scoping review. An expert commentary was included in the editorial.

### Geographic distribution of scientific production

The studies were conducted in 22 countries, with a predominance in the United States (n = 7), followed by England (n = 4), Australia (n = 3), Singapore (n = 3), Belgium (n = 2), and South Korea (n = 2). In alphabetical order, the remaining 16 countries identified in this selection, each with one study, were: Saudi Arabia, Argentina, Austria, Brazil, Chile, Spain, Finland, Hong Kong, India, Iran, Ireland, Israel, Italy, Nigeria, Sweden, and Tunisia.

Europe, represented by England, Austria, Belgium, Spain, Finland, Ireland, Italy, and Sweden, was represented by 12 studies, followed by the Americas, with 10 studies from the United States, Brazil, Chile, and Argentina. Next was Asia, with studies from Singapore, South Korea, Saudi Arabia, Hong Kong, India, Iran, and Israel, presenting 10 eligible studies. Oceania was represented by Australia, with three studies, and Africa by Nigeria and Tunisia, with two studies.

### Distribution of the professional categories studied

The selected studies included the following combinations of professionals: health professionals without specifying profession (n = 14), physicians (n = 9), nurses (n = 7), physicians and nurses in the same study (n = 3), service providers (n = 1), physicians, nurses, and midwives (n = 1), nurses and midwives (n = 1), and nursing staff (n = 1).

### Care settings associated with the phenomenon

A predominance of hospitals was identified (n = 25), followed by studies in which no reference was made to the setting (n = 7), studies that described various settings (n=4), and one study with a mixed setting involving hospitals and health centers.

Of the studies conducted in hospitals, 18 did not specify a particular department; three were in the intensive care unit, two in the operating room, one in the trauma center, and one in the inpatient ward.

### Distribution of publications over time

**Fig 2:**
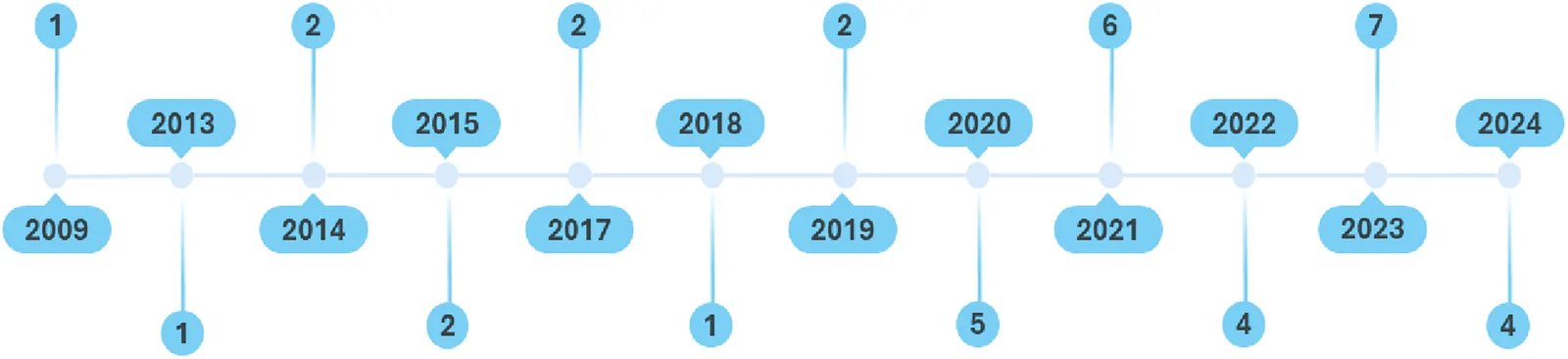
Temporal distribution of records included in the scoping review. Rio de Janeiro, RJ, Brazil, 2025. Elaborated by authors, 2025.

The analyzed publications spanned the period from 2009 to 2024, with the highest concentration in the years 2023 (n = 7), 2021 (n = 6), 2020 (n = 5), 2024 (n = 4), and 2022 (n = 4). Next, the years 2014, 2015, 2017, and 2019 each had two studies. Finally, in the years 2009, 2013, and 2018, one study was identified in each year, as illustrated in Fig 2.

### Instruments used to identify the second victim phenomenon

In identifying the repercussions resulting from the second victim phenomenon among the 37 studies included in this review, 13 studies used measurement scales. The scales were: *PTSD Checklist for DSM-5* (PCL-5 scale) [33,34], Impact of Event Scale – Revised (IES-R) [41,43], *Second Victim Experience and Support Tool* (SVEST) [29,37,42,48,55], Healthcare Professional Error Experience Questionnaire (HPEEQ) [24], Post-Traumatic Stress Disorder Screening in Primary Care for *DSM-5* (PC-PTSD-5 [50]), Suicide Behaviors Questionnaire – Revised (SBQ-R) [40], *Second Victim Experiences and Distress – International Survey* (SeViD-I Survey) [49].

The remaining studies used structured interviews organized by the authors themselves or did not mention the method used to identify the repercussions.

### Analysis of health descriptors used in studies on the second victim phenomenon

To assess the relevance of the studies in relation to the objective of the scoping review, the frequency of use of descriptors and keywords in the publications was analyzed. The identified keywords reinforce the alignment of studies on the second victim phenomenon within the scope of patient safety, although they were also found, albeit to a limited extent, in publications focused on worker safety and protection.

Of the 64 descriptors or keywords identified, those with the highest frequency were: second victim (n = 17), adverse events (n = 14), patient safety (n = 8), healthcare professionals (n = 7), medical error (n = 5), and nursing (n = 4).

Although still in its early stages, the use of the terms “leaving the profession” (n=1), “absenteeism” (n=1), “well-being at work” (n=1), “intention to rotate” (n=1), “mental health” (n=2), “post-traumatic bitterness disorder” (n=1), “post-traumatic stress disorder” (n=1), “suffering” (n=2), “suicide” (n=1), and “support program” (n=2), which are clearly affiliated with the field of work and occupational health and safety.

### Repercussions arising from the second victim phenomenon

From the manifestations of more enduring signs and symptoms affecting professionals in the medium and long term, 39 repercussions emerged, which are listed in Table 3.

**Table 3:** Characterization of the repercussions identified in the review. Rio de Janeiro, RJ, Brazil, 2025.

Categorization of Repercussions	Mapping the repercussions
**Dimension** **Physics**	Increase in alcohol use [36,37,43,45]
	Increase in substance use [36,45]
**Dimension** **Mental**	Suicidal thinking or behavior [23,36,40,45,46,50] Post-traumatic bitterness disorder (PTED) [23,31,35,44]
	Post-traumatic stress disorder (PTSD) [23,24,31,35,44,48,53]
	Decrease or reduction in job satisfaction [30,39,41,51,53,54,56]
	Repetitive/intrusive/disturbing/recurring/flashback memories [25,27,29,34,35,47]
	Burnout syndrome [46]
	Concentration problems [42.43]
	Hypervigilance/Overly Cautious [34,44,49,53]
**Dimension** **Professional**	Away from the workplace/taking time off [22,40,45]
	Threat to professional identity [26,30]
	Early retirement [6]
	Doubts about the knowledge and ability/feeling of inability to provide quality care/professional incompetence/decline in job performance/negatively affected job performance [26,33–35,37,39,49,53]
	Intention to turnover, change of function and job [6,28,33,38,40,45]
	Vigilance in relation to errors/new insights/patient safety advocates/motivation for improvement/sought learning/attention to detail/change in practice positively/learning/more cautious/reflective and aware to improve/risk-averse/working calmly [25,26,29,32,49,56]
	Change/abandonment of profession, change of specialty, rethink career [6,21,22,26,32,33,36,44,45,47,51,52,56]
	Job loss and financial consequences [21,32,51,52]
	Defensive practice [56]
	Judicial Process/Fear of Disciplinary Action/Punitive Process/Loss of Professional Registration [21,30,32,37,51–53,56]
	Impaired professional relationships [43,49,54]
	Reputation affected/labeled as incompetent or careless by colleagues, their family, and the patient's family/loss of respect from co-workers [21,39,41,56]
	Aggressive behavior [42]
	Positive Practice Change/Learning/Better Vigilance Regarding Mistakes [25,26,29,32,49,56]
	Suboptimal performance [46,47,49,51,53,54]
	Reduced or Loss of Confidence [46,47,49,51,53,54]
	Resilience [55]
	Inability to move on/event will remain for career [6,26,47]
**Dimension** **Social**	Change in leisure activities [32]
	Threat to personal identity/depersonalization [30]
	Difficulty in personal life/difficulty interacting with family members/adverse effect on family life [33]
	Self-doubt [34–36,47]
	Stigmatization [26]
	Excessive excitability/internal inadequacy/change in behavior [24]
	Social isolation/loneliness [26,39,47]
	Reactivity in places outside of work [42]
	Reduction in quality of life [34]
	Damaged personal relationships [43,51,56]
	Sense of damaged personal integrity [46]

Elaborated by authors, 2025.

These repercussions were analyzed and categorized in light of worker health, using the theoretical assumptions of the Psychodynamics of Work Theory [57] and the Theory of Social Determinants [58], and were grouped into four dimensions.

The physical dimension, based on four studies, presented two repercussions indicating a risk related to increased consumption of alcohol and other substances, suggesting a risk to the physical integrity of professionals [36,37,43,45].

The mental dimension depicted eight repercussions presented in 22 studies and described as “repetitive,” “disturbing,” and “intrusive” [25,27,29,34,35,47], combined with “post-traumatic grief disorder,” “post-traumatic stress disorder,” and “burnout syndrome” [23,24,31,35,44,48,53]. In this dimension, one of the most severe repercussions was “suicidal thoughts or behavior” [33,36,45,46,50].

In the professional dimension, 18 repercussions identified in 30 studies were grouped together; this was the dimension encompassing the largest number of studies in this review, demonstrating the phenomenon’s strong impact on the work activities of those involved.

Notable in the professional dimension are the repercussions described as “change of profession,” “leaving the profession,” “change of specialty,” and “rethinking one’s career” [6,21,22,26,32,33,36,44,45,47,51,52,56].

Last but not least, 15 studies were grouped under the social dimension, which depicted 11 repercussions with the potential to alter the professional’s life outside the workplace. In this dimension, “self-doubt” [34–36,47], “isolation” [26,39,47], and “impaired personal relationships” [43,51,56] stood out.

Despite the deleterious effect of the second victim phenomenon, six studies grounded in safety culture elements described “vigilance regarding errors” and “positive practice change” as repercussions with a positive impact [25,26,29,32,49,56].

## Discussion

The “second victim” phenomenon has been consistently discussed in developed countries [6,19,21,22,32,33,38,39,42,44,47,50,51,54] and is gaining visibility, albeit modestly, in developing countries [29,43,45,46,48,49,52,53].

Although it is discussed in these developing countries and scarce in some regions [24,43], it is observed that the 37 studies identified in the review covered 22 are distributed in countries on five continents, which points to a similar manifestation of the phenomenon, regardless of culture [31].

The issue of patient and second victim safety can be contextualized and analyzed from different perspectives, with the provision of safe and quality care as its central axis [1,2,8,10]. In this context, the relationship between the patient and the health professional is highlighted, considering the perceptions that permeate this binomial [2,7,57–61]. This approach also calls for researchers in the field of work to reflect on care practices, their implications for the professionals involved, and their impacts on the quality of care.

Although the term “second victim” has been contested [8], the phenomenon affects, impacts, and has consequences for professionals in one of the layers that underpin the social determinants of health, namely the workplace [9,57–62]. The review spanned 15 years, with the first study identified in 2009 and the most recent in 2024, the year prior to the database search. It is observed that between 2020 and 2024 there was an increase in publications on the phenomenon of the second victim, with 26 studies in four years compared to the first 10 years, during which 11 studies were identified. This increase in publications began in 2020, when profound transformations occurred globally in the world of work, especially for healthcare professionals, as a result of the COVID-19 pandemic [1], contributing to discussions on the topic.

Considering the magnitude of work as a guarantee of life—which, beyond means and conditions of subsistence, implies social identity and belonging—the identified repercussions point to the legitimacy of analyzing the phenomenon from a work-centered perspective [57,58,60,62,63].

The studies presented in this review indicate that naming and conceptualizing professional suffering resulting from involvement in safety incidents is a sound decision for developing policies to identify manifestations and repercussions, as well as for creating support programs [1,2,5,6,10,57,58,61,62].

The safety, quality, and safe care policies advocated by the World Health Organization and the International Labour Organization [62] cut across the work of healthcare professionals and seek to address the barriers that hinder the understanding that worker safety is a pillar for ensuring patient safety [1,2,6,57,58,61,64].

Although the review of the scientific evidence encompassed healthcare professionals across various disciplines, the nursing profession was the professional group most affected by the phenomenon, with this review identifying 27 studies in which nursing was among the population studied, nine of which were conducted exclusively with nurses [28,33,36,37,40,44,47,48,52].

Being the most cited category, nursing had repercussions across all categorized dimensions, particularly in the professional dimension, where the repercussions “leaving the profession,” “intention to leave,” “changing jobs,” and “changing professions” [26,31,34,35,44,49] generate impacts not only on the lives of these professionals but also on healthcare organizations, by latently triggering global concern regarding the organization of work in healthcare systems as it pertains to nursing services [57–59,65]. The prevalence of nursing can be discussed based on the characteristics of nursing work that can influence the occurrence of these incidents, which are more susceptible to the phenomenon [66,67].

A systematic review of the literature identified that women tend to report significantly more distress than men [26]. However, regardless of professional category or gender, health professionals' responses to safety incidents "are greater than mere emotional reaction" [24,29]. Physicians and nurses when they are mentally ill are more likely to be involved in patient safety incidents, leading to potential harm and even death to patients [65,68,69].

The manifestations of the second victim phenomenon do not have specific clinical criteria [47]. They encompass a range of symptoms and feelings that lead to catastrophic changes with the potential to cause physical and mental illness and pose a risk to the well-being of those involved [2,6,10,19,53].

The risk of suicide among healthcare professionals was highlighted during the COVID-19 pandemic, as it revealed the levels of psychological stress to which professionals were subjected due to high workloads and long hours, during which they lived in constant fear of exposure to the disease [1]. However, in the Health Workers’ Safety Charter, the WHO notes that health professionals already faced a higher risk of suicide worldwide, corroborating the studies reviewed here that identify suicide risk as a major concern regarding worker health [1,23,36,40,45,46,50]. A study published in 2021 reports that the methods and samples used regarding the second victim do not intentionally capture the risk of suicide, which certainly represents a serious shortcoming for all those affected by the suicide of a second victim [40].

When analyzing the second victim phenomenon through the lens of this study, it becomes clear that when institutional culture does not align with current just culture recommendations, harm to the professional is inevitable, altering their work relationship and sense of belonging to the social group in which they are embedded—which generates another layer of suffering beyond the characteristic manifestations of the second victim phenomenon already described [55,57–60,62].

In addition to the symptoms already identified in the literature [2,6,8,11,63] there is a need for investment in tools for the early detection of distress among these professionals, so that specialized care and intervention strategies can be provided; these strategies, in addition to fostering a just culture, should ensure physical, mental, social, and financial protection for those involved in patient safety incidents [8,31,32,37,39,46,47].

Given the prevalence of nursing in the analyzed studies, the “State of the World’s Nursing” report warns of a decline in the training and retention of nurses in the labor market and identifies as causes of demotivation for enrolling in nursing training programs, inadequate working conditions, the absence of specific policies regarding nursing staff work hours, job insecurity, and the lack of mental health and well-being programs [65,70].

Still on the topic of work, PAHO expresses a latent concern regarding the shortage of health professionals and the organization of work in health systems globally [70], it appears that the identified repercussions [6,21,56] directly affect this workforce regarding leaving the profession, changing jobs, defensive practice, and mental health issues [57–62].

In line with the PAHO report [70], studies identified in this review warned that professionals suffering from the “second victim” phenomenon experience a transactional model of experience, shaped by safety culture and external influences [36,54,55], corroborating the theory of pleasure and suffering arising from work [58].

Concerns about the impact of the “second victim” phenomenon on healthcare professionals align with concerns regarding the organization of work in the healthcare sector [62] and the protection of professionals through the development and implementation of safe healthcare systems [2], as supported by Strategic Objective 5 of the Global Patient Safety Action Plan 2021–2030 [2] and Goal 8 of the Sustainable Development Goals [61].

European researchers have pointed out weaknesses in the process of raising awareness about the second victim phenomenon, particularly in the training of healthcare professionals [71], noting that the topic is neglected in medical and nursing curricula at European universities and that, given the frequency with which these events occur, preparing students to cope with the effects of such events when they enter the workforce is a fundamental step toward identifying the phenomenon and strengthening protective barriers to prevent illness [65,70–72].

The WHO, which understands that governments and institutions have a legal and moral responsibility to ensure the health, safety, and well-being of healthcare professionals, links the guarantee of patient safety to improvements in professional safety and reinforces that mental health and work are inextricably linked [1,55,57–60,62], it is therefore recommended that healthcare institutions adopt measures to support those involved, in order to prevent the outcomes identified in the dimensions categorized in this study.

Finally, the review highlights the lack of relevance of the descriptors or keywords used to index studies on the second victim phenomenon in the workplace, which corroborates the curricular shortcomings identified in other studies [68,70,71], contributing to the topic’s divergence from the recommendations of the WHO, ILO, and PAHO regarding the convergence between safe care and worker safety [1–3,11,61,62,70].

### Study limitations

A notable limitation is that the term “second victim” is not a structured term in the Medical Subject Headings, leading to semantic variability and the use of different expressions, such as second victim phenomenon, second victim syndrome, and second victim experience.

Another point is the characterization of manifestations in studies addressing the second victim phenomenon without distinguishing between acute and chronic manifestations, which may lead to an imprecise understanding of the temporal nature of outcomes.

A lack of specification regarding healthcare settings and services was also observed in some of the analyzed publications. Given that different care environments have distinct organizational characteristics, work processes, and workloads, this lack of detail limits the understanding of the particularities that may influence the repercussions of the second victim phenomenon.

Additionally, there was a low representation of studies involving professional categories beyond medicine and nursing, highlighting the need to expand investigations to other professions and to healthcare workers in general, including support staff working in healthcare services.

Finally, it should be noted that, as this is a scoping review, the study aimed to map and synthesize the available evidence on the topic without conducting a critical assessment of the methodological quality of the included studies.

### Contributions of the study

This scoping review synthesized the evidence on the repercussions of the second victim phenomenon on health professionals, organizing the findings in the light of the references of the Psychodynamics of Work and the Social Determinants of Health, which enabled a broader understanding of the impacts of this phenomenon on workers. The identification of serious repercussions, such as suicidal ideation, reinforces the need for institutional strategies aimed at welcoming, monitoring, and supporting professionals involved in adverse events. Institutions should invest in tools for early identification of illness and in specific support programs in the workplace, which is essential to mitigate the repercussions, strengthening the resilience of health professionals in the face of the challenges inherent to clinical practice.

## Conclusion

The mapping of the repercussions experienced by healthcare professionals who undergo the “second victim” phenomenon resulting from patient safety incidents in healthcare services revealed manifestations with long-lasting signs and symptoms that affect professionals in the medium and long term.

In the 37 selected studies, the review identified 39 repercussions affecting professionals in the physical, mental, professional and social dimensions. Of the identified repercussions, only two have the potential for positive manifestations, while the remaining manifestations are detrimental to the professional, such as suicidal ideation, which was identified in the mental dimension and which, despite being little discussed in the studies of this review, represents a serious threat to the physical integrity of professionals suffering from the second victim phenomenon.

The professional dimension encompassed the largest number of studies and repercussions, indicating that the phenomenon has a strong connection to the fields of work, occupational health, and occupational safety.

Despite the potential for implications in the professional sphere, the analysis of the descriptors indicated that the publications are strongly linked to the field of patient safety and distant from the field of labor.
